# Prevalence and Characteristics of Subjective Cognitive Decline Among Unpaid Caregivers Aged ≥45 Years — 22 States, 2015–2019

**DOI:** 10.15585/mmwr.mm7046a1

**Published:** 2021-11-19

**Authors:** Eva M. Jeffers, Erin D. Bouldin, Lisa C. McGuire, Kenneth A. Knapp, Roshni Patel, Dana Guglielmo, Christopher A. Taylor, Janet B. Croft

**Affiliations:** ^1^Division of Population Health, National Center for Chronic Disease Prevention and Health Promotion, CDC; ^2^Oak Ridge Institute for Science and Education, Oak Ridge, Tennessee; ^3^Department of Health and Exercise Science, Appalachian State University, Boone, North Carolina; ^4^Department of Public Health and Center for Long-Term Care, New York Medical College, Valhalla, New York; ^5^CyberData Technologies, Inc., Herndon, Virginia.

Approximately 20% of U.S. adults are unpaid caregivers (caregivers) (*1*) who provide support to a family member or friend with a health condition or disability. Although there are benefits to caregiving, it can negatively affect caregivers’ physical and mental health (*2*–*4*). Much of the assistance caregivers provide, such as administering medications or financial management, relies on cognitive ability, but little is known about caregivers’ cognitive functioning. Subjective cognitive decline (SCD), the self-reported experience of worsening or more frequent confusion or memory loss over the past year (*5*), could affect caregivers’ risk for adverse health outcomes and affect the quality of care they provide. CDC analyzed SCD among caregivers aged ≥45 years through a cross-sectional analysis of data from 22 states in the 2015−2019 Behavioral Risk Factor Surveillance System (BRFSS). Among adults aged ≥45 years, SCD was reported by 12.6% of caregivers who provided care to a family member or friend with a health condition or disability in the past 30 days compared with 10.2% of noncaregivers (p<0.001). Caregivers with SCD were more likely to be employed, men, aged 45–64 years, and have chronic health conditions than were noncaregivers with SCD. Caregivers with SCD were more likely to report frequent mental distress, a history of depression, and frequent activity limitations than were caregivers without SCD. SCD among caregivers could adversely affect the quality of care provided to care recipients. Understanding caregivers’ cognitive health and the types of care provided is critical to maintaining the health, well-being, and independence of the caregiving dyad. Health care professionals can support patients and their patients’ caregivers by increasing awareness among caregivers of the need to monitor their own health. The health care team can work with caregivers to identify potential treatments and access supports that might help them in their caregiving role and compensate for SCD.*

BRFSS is a cross-sectional, random-digit–dialed, annual telephone survey of noninstitutionalized U.S. adults aged ≥18 years. BRFSS is conducted by state and territorial health departments, and data are weighted to make estimates representative of each state. Combined (landline and mobile) median response rates for 2015−2019 ranged from 45.9% (2017) to 49.9% (2018).^†^ Among 22 states^§^ in which BRFSS respondents were asked both the caregiving and cognitive decline questions in the same survey year during 2015−2019, the most recent year’s data were analyzed for this study.

Respondents were classified as caregivers if they responded affirmatively when asked whether they had provided care to a family member or friend with a health condition or disability in the past 30 days. These respondents were then asked seven more questions about the care recipient and the type and duration of care provided (*1*). Personal care tasks included administering medications, feeding, dressing, and bathing; household tasks included cleaning, managing money, and preparing meals.^¶^ Respondents were classified as experiencing SCD if they responded affirmatively when asked if they had experienced worsening or more frequent confusion or memory loss in the past 12 months.

Weighted, unadjusted prevalence of SCD by caregiver status was estimated among 93,604 community-dwelling respondents aged ≥45 years and among a subgroup of 21,238 (23.0%) caregivers, by sociodemographic, health-related, and caregiving-related characteristics. The distribution of these characteristics was estimated among caregivers by SCD status. Complex survey data methods were used to estimate weighted percentages and corresponding 95% CIs using SAS-callable SUDAAN survey procedures (version 9.4; SAS Institute). T-tests were used to determine statistically significant differences between caregivers and noncaregivers with SCD, and modified Rao-Scott chi-square tests were used to estimate statistical differences between proportions of caregivers with and without SCD for each selected characteristic. P-values <0.05 were considered statistically significant for both tests. The relative standard error for all estimates was <30%. This activity was reviewed by CDC and was conducted consistent with applicable federal law and CDC policy.**

During 2015–2019, 23.0% (95% CI = 22.5%–23.6%) of U.S. adults (approximately 13 million) aged ≥45 years in 22 states were caregivers. Among caregivers, the overall prevalence of SCD was 12.6% and varied by state, ranging from 9.8% (New Jersey) to 17.3% (Louisiana) (Table 1). In comparison, the prevalence of SCD among noncaregivers was 10.2% (p<0.001) (Table 2). Prevalence of SCD did not differ between caregivers and noncaregivers by history of depression, number of days physical or mental health was not good in the past 30 days, or number of days health prevented regular activities in the past 30 days. Compared with noncaregivers, SCD prevalence among caregivers was higher among persons aged 45–64 years, men, non-Hispanic White persons, employed persons, persons who reported any chronic condition, and persons who reported good, very good, or excellent health.

**TABLE 1:** Prevalence of subjective cognitive decline* among unpaid adult caregivers^†^ aged ≥45 years, by state — Behavioral Risk Factor Surveillance System, 22 states,^§^ 2015–2019

State	No. of respondents who are caregivers	Estimated (weighted)^¶^ no. of caregivers	Weighted^¶^ % with SCD (95% CI)
**Overall**	**21,238**	**12,693,000**	**12.6 (11.7**–**13.5)**
Alabama	1,257	465,000	14.1 (11.6–16.7)
Florida	683	1,864,000	14.6 (11.2–18.0)
Hawaii	898	99,000	12.0 (9.3–14.7)
Illinois	665	975,000	10.1 (7.5–12.6)
Iowa	692	191,000	10.6 (7.9–13.2)
Louisiana	736	415,000	17.3 (13.8–20.8)
Maryland	1,029	585,000	11.8 (9.3–14.3)
Mississippi	934	248,000	15.7 (12.4–18.9)
Missouri	913	464,000	15.5 (11.6–19.3)
Montana	734	78,000	13.5 (9.8–17.1)
Nebraska	1,510	181,000	11.3 (8.9–13.8)
New Jersey	396	720,000	9.8 (5.3–14.3)
New York	634	1,482,000	11.1 (7.6–14.5)
Oregon	783	355,000	13.7 (10.3–17.0)
South Carolina	1,716	430,000	15.3 (12.9–17.6)
Tennessee	1,005	664,000	12.7 (10.1–15.3)
Texas	1,767	1,877,000	11.8 (9.3–14.2)
Utah	798	234,000	14.9 (11.8–17.8)
Virginia	1,434	704,000	11.0 (9.0–13.0)
West Virginia	948	195,000	11.1 (8.7–13.5)
Wisconsin	765	413,000	14.3 (10.8–17.8)
Wyoming	941	54,000	11.9 (9.0–14.9)

**Abbreviation:** SCD = subjective cognitive decline.

* SCD was defined as the self-reported experience of worsening confusion or memory loss in the past year.

^†^ Caregiving was defined as providing care to a family member or friend with a health condition or disability in the past 30 days.

^§^ The following 22 U.S. states that included both caregiving and SCD modules in the same survey year during 2015–2019 are included (most recent year used): Alabama (2015), Florida (2015), Hawaii (2017), Illinois (2015), Iowa (2015), Louisiana (2015), Maryland (2019), Mississippi (2015), Missouri (2016), Montana (2016), Nebraska (2015), New Jersey (2018), New York (2019), Oregon (2019), South Carolina (2015), Tennessee (2019), Texas (2019), Utah (2019), Virginia (2019), West Virginia (2015), Wisconsin (2015), and Wyoming (2015).

^¶^ Estimates are weighted to each state’s adult population.

**TABLE 2:** Percentage of subjective cognitive decline* among unpaid caregivers^†^ and noncaregivers aged **≥**45 years, by selected characteristics — Behavioral Risk Factor Surveillance System, 22 states,**^§^** 2015–2019

Characteristic	Total unweighted no. of caregivers^¶^	Caregivers with SCD, weighted** % (95% CI)	Total unweighted no. of noncaregivers^¶^	Noncaregivers with SCD, weighted** % (95% CI)	p-value^††^
**Overall**	**21,238**	**12.6 (11.7–13.5)**	**72,366**	**10.2 (9.7–10.7)**	**<0.001**
**Demographic characteristic**					
**Age group, yrs**					
45–64	12,049	12.4 (11.3–13.6)	34,858	9.4 (8.8–10.0)	<0.001
≥65	9,189	13.0 (11.7–14.4)	37,508	11.4 (10.7–12.1)	0.03
**Sex**					
Men	7,615	13.5 (12.1–15.0)	31,370	9.4 (8.8–10.1)	<0.001
Women	13,623	12.0 (10.9–13.2)	40,993	10.9 (10.2–11.6)	0.09
**Race/Ethnicity**					
White, non-Hispanic	16,689	12.9 (11.9–13.9)	56,555	9.7 (9.2–10.2)	<0.001
Black, non-Hispanic	2,226	12.0 (9.5–14.5)	7,184	12.7 (11.1–14.3)	0.6
Asian/Pacific Islander, American Indian/Alaska Native, Other race/Multiracial, non-Hispanic^§§^	1,312	14.3 (8.9–19.8)	4,476	9.9 (7.9–11.9)	0.1
Hispanic	673	9.6 (6.2–13.0)	2,963	10.8 (8.9–12.7)	0.6
**Education level**					
High school graduate or less	7,041	15.0 (13.3–16.6)	27,920	7.9 (7.4–8.3)	0.06
Some college or more	14,160	11.3 (10.3–12.3)	44,217	13.2 (12.4–14.1)	<0.001
**Employment status**					
Employed/Self-employed	8,933	7.6 (6.5–8.7)	27,914	4.8 (4.3–5.2)	<0.001
Unemployed	829	21.0 (14.6–27.4)	1,994	14.8 (11.6–18.0)	0.09
Unable to work	1,900	37.2 (33.1–41.4)	6,869	31.5 (29.1–33.8)	0.01
Other^¶¶^	9,460	11.5 (10.3–12.6)	35,185	10.4 (9.7–11.1)	0.1
**Health-related characteristic**					
Any chronic condition***					
Yes	14,302	16.4 (15.1–17.6)	47,206	13.4 (13.1–14.5)	<0.001
No	6,777	5.7 (4.6–6.7)	24,620	4.3 (3.8–4.8)	0.02
**History of depression**					
Yes	4,915	28.3 (25.8–30.8)	12,582	27.2 (25.9–29.4)	0.7
No	16,239	8.0 (7.2–8.9)	59,462	6.8 (6.4–7.2)	0.01
**General health status**					
Good, very good, or excellent	16,454	8.1 (7.4–9.0)	55,300	6.1 (5.7–6.5)	<0.001
Fair or poor	4,734	26.8 (24.3–29.4)	16,855	23.3 (21.9–24.8)	0.02
**No. of days physical health was not good in past 30 days**					
None	12,106	6.5 (5.7–7.4)	43,638	5.1 (4.7–5.5)	0.003
1–13	5,196	15.2 (13.2–17.1)	14,985	13.2 (12.0–14.5)	0.09
≥14	3,550	28.2 (25.2–31.1)	11,878	24.7 (23.0–26.2)	0.04
**No. of days mental health was not good in past 30 days**					
None	13,363	6.2 (5.5–6.9)	52,915	5.5 (5.1–5.9)	0.1
1–13	4,692	16.1 (14.0–18.1)	11,691	14.7 (13.4–16.0)	0.3
≥14	2,853	34.4 (30.7–38.0)	6,424	36.5 (33.9–39.2)	0.3
**No. of days health prevented regular activities in past 30 days**					
None	6,469	11.4 (9.8–13.0)	18,832	9.5 (8.7–10.3)	0.03
1–13	3,159	19.0 (16.4–21.7)	8,211	16.5 (14.7–18.2)	0.1
≥14	2,340	33.9 (30.1–37.8)	7,615	33.8 (31.4–36.2)	0.9

**Abbreviation:** SCD = subjective cognitive decline.

* SCD was defined as the self-reported experience of worsening confusion or memory loss in the past year.

^†^ Caregiving was defined as providing care to a family member or friend with a health condition or disability in the past 30 days.

^§^ The following 22 U.S. states that included both caregiving and SCD modules in the same survey year during 2015–2019 are included (most recent year used): Alabama (2015), Florida (2015), Hawaii (2017), Illinois (2015), Iowa (2015), Louisiana (2015), Maryland (2019), Mississippi (2015), Missouri (2016), Montana (2016), Nebraska (2015), New Jersey (2018), New York (2019), Oregon (2019), South Carolina (2015), Tennessee (2019), Texas (2019), Utah (2019), Virginia (2019), West Virginia (2015), Wisconsin (2015), and Wyoming (2015).

^¶^ Categories might not sum to the sample total because of missing responses.

** Estimates are weighted to each state’s adult population.

^††^ T-tests were used to determine statistically significant differences between caregivers and noncaregivers with SCD for each level of selected characteristics at p<0.05.

^§§^ Asian/Pacific Islander, American Indian/Alaska Native, and Other or multiracial non-Hispanic persons were combined into one group because of small sample sizes.

^¶¶^ Homemaker, student, or retired.

*** Any chronic condition was determined by an affirmative response to the question, “Has a doctor or other health professional ever told you that you had any of the following? For each, tell me Yes, No, or You’re Not Sure: asthma (current); heart attack, angina, or coronary heart disease; a stroke; cancer other than skin cancer; chronic obstructive pulmonary disease, emphysema, or chronic bronchitis; some form of arthritis, rheumatoid arthritis, gout, lupus, or fibromyalgia; kidney disease, not including kidney stones, bladder infections, or incontinence; or diabetes, not including gestational, borderline, or prediabetes?”

The distribution of caregiver characteristics varied by SCD status (Table 3). Compared with caregivers without SCD, those with SCD were more likely to have at least one chronic condition,^††^ a history of depression, report fair or poor health, report ≥14 days of poor physical health in the past 30 days, report ≥14 days of poor mental health in the past 30 days, and report ≥14 days that health prevented regular activities in the past 30 days. Household status, duration or type of care provided, or the care recipient having a diagnosis of Alzheimer’s disease, dementia, or other cognitive impairment disorder did not differ by SCD status.

**TABLE 3:** Distribution of selected characteristics among unpaid caregivers* aged **≥**45 years by subjective cognitive decline status^†^ — Behavioral Risk Factor Surveillance System, 22 states,**^§^** 2015–2019

Characteristic	Caregivers, weighted^¶^ % (95% CI)		p-value**
	With SCD (n = 2,670)	Without SCD (n = 18,568)	
**Household status**			
Lives alone	20.0 (17.0–23.0)	17.6 (16.5–18.90	0.1
Does not live alone	80.0 (77.0–83.0)	82.4 (81.4–83.5)	
**Health-related characteristic**			
**Any chronic condition** ^††^			
Yes	84.7 (82.0–87.3)	62.9 (61.4–64.4)	<0.001
No	15.3 (12.7–18.0)	37.1 (35.6–38.6)	
**History of depression**			
Yes	50.3 (46.6–54.0)	18.3 (17.1–19.4)	<0.001
No	49.7 (46.0–53.4)	81.7 (80.6–82.9)	
**General health status**			
Good, very good, or excellent	49.3 (45.6–53.0)	80.0 (78.7–81.2)	<0.001
Fair or poor	50.7 (47.0–54.4)	20.0 (18.8–21.3)	
**No. of days physical health was not good in past 30 days**			
None	29.2 (25.8–32.5)	60.2 (58.6–61.7)	<0.001
1–13	31.7 (28.1–35.2)	25.5 (24.1–26.8)	
≥14	39.2 (35.5–42.8)	14.4 (13.3–15.5)	
**No. of days mental health was not good in past 30 days**			
None	30.0 (26.9–33.2)	66.0 (64.5–67.5)	<0.001
1–13	30.7 (27.2–34.1)	23.2 (21.8–24.5)	
≥14	39.3 (35.5–43.1)	10.8 (9.9–11.8)	
**No. of days health prevented regular activities in past 30 days**			
None	33.8 (29.8–37.7)	57.3 (55.2–59.3)	<0.001
1–13	29.0 (25.3–32.6)	26.9 (25.0–28.8)	
≥14	37.3 (33.4–41.2)	15.8 (14.2–17.4)	
**Main health condition of care recipient**			
Alzheimer’s disease/Cognitive impairment/Dementia	12.3 (10.0–14.6)	12.8 (11.8–13.8)	0.7
All other health conditions	87.7 (85.4–90.0)	87.2 (86.2–88.2)	
**Length of care provided, yrs**			
<5	66.3 (62.8–69.7)	67.6 (66.0–69.2)	0.5
≥5	33.7 (30.3–37.2)	32.4 (30.8–87.0)	
**No. of weekly hours of care provided**			
<20	65.0 (61.3–68.8)	68.7 (67.2–70.2)	0.08
≥20	35.0 (31.2–38.7)	31.3 (29.8–32.8)	
**Type of assistance provided**			
Personal care only^§§^	7.9 (5.7–10.1)	6.9 (6.0–7.8)	0.8
Household tasks only^¶¶^	37.6 (33.9–41.3)	38.5 (36.9–40.2)	
Personal care and household tasks	54.5 (50.6–58.4)	54.6 (52.9–56.3)	
Neither personal care nor household tasks	19.0 (15.8–22.3)	18.0 (16.8–19.2)	

**Abbreviation:** SCD = subjective cognitive decline.

* Caregiving was defined as providing care to a family member or friend with a health condition or disability in the past 30 days.

^†^ SCD was defined as the self-reported experience of worsening confusion or memory loss in the past year.

^§^ The following 22 U.S. states that included both caregiving and SCD modules in the same survey year during 2015–2019 are included (most recent year used): Alabama (2015), Florida (2015), Hawaii (2017), Illinois (2015), Iowa (2015), Louisiana (2015), Maryland (2019), Mississippi (2015), Missouri (2016), Montana (2016), Nebraska (2015), New Jersey (2018), New York (2019), Oregon (2019), South Carolina (2015), Tennessee (2019), Texas (2019), Utah (2019), Virginia (2019), West Virginia (2015), Wisconsin (2015), and Wyoming (2015).

^¶^ Estimates are weighted to each state’s adult population.

** P-values from chi-square analyses measure the association between proportions, with modified Rao-Scott chi-square tests.

^††^ Any chronic condition was determined by an affirmative response to the question, “Has a doctor or other health professional ever told you that you had any of the following? For each, tell me Yes, No, or You’re Not Sure: asthma (current); heart attack, angina, or coronary heart disease; a stroke; cancer other than skin cancer; chronic obstructive pulmonary disease, emphysema, or chronic bronchitis; some form of arthritis, rheumatoid arthritis, gout, lupus, or fibromyalgia; kidney disease, not including kidney stones, bladder infections, or incontinence; or diabetes, not including gestational, borderline, or prediabetes?”

^§§^ Personal care tasks were defined as administering medications, feeding, dressing, or bathing.

^¶¶^ Household tasks were defined as cleaning, managing money, or preparing meals.

## Discussion

Among caregivers aged ≥45 years in 22 participating states, approximately one in eight reported SCD, the self-reported experience of worsening or more frequent confusion or memory loss over the past year. SCD was more common in caregivers than in noncaregivers, particularly among those aged 45–64 years. SCD likely affects the quality and safety of care that caregivers can provide. Caregivers with SCD more frequently experienced negative physical and mental health than did caregivers without SCD. In addition, caregivers with SCD more frequently reported chronic conditions, being employed, being men, and were younger than noncaregivers with SCD, suggesting specific opportunities for interventions among caregivers with SCD. These findings are consistent with studies that indicate that, although there are benefits to caregiving, it can negatively affect a caregiver’s physical and mental health (*2*–*4*). Adverse health outcomes have been found to be related to physical, emotional, and financial strains placed on caregivers, prioritization of care recipients’ needs over caregivers’ needs, and changes in behaviors that support caregivers’ health such as delaying medical care or decreased physical activity (*2*–*4*).

As the U.S. population continues to age (*6*), the number of persons needing care is expected to increase. SCD among caregivers might make it more difficult to help care recipients manage medications, finances, or other aspects of their chronic conditions or health needs that require cognitive focus. Whether a caregiver with SCD can provide the level of support that is needed, and if so, for how long, are important considerations. Limitations in functional activities because of SCD might result in the need for assistance (*5*). Caregivers might need support themselves, both currently and in the future, especially given that this study found that more caregivers than noncaregivers experience SCD. SCD might be a symptom of early-stage dementia or a sign that more serious cognitive decline will occur in the future. SCD might also be a result of other health conditions that could be treatable, such as infections, medication interactions, or nutritional deficits, and potentially remain stable over time (*7*,*8*). Caregivers are a crucial component of a caregiving team; however, these data suggest that caregivers, particularly those with SCD, might need support for their own health and well-being challenges. 

The findings in this report are subject to at least four limitations. First, causality between caregiving and SCD cannot be inferred from a cross-sectional study. Second, self-reported data might be subject to several biases, including recall and social desirability biases, which might result in under- or overreporting of SCD. Third, these data cannot be validated with medical examination records, but the perception of decline (versus objectively measured decline) is associated with development of Alzheimer’s disease or other dementias (*9*,*10*). Finally, with data from 22 states, the findings of this report cannot be extrapolated to the rest of the country. A major strength of this study is the large sample size of caregivers.

Considering the growth of the older adult population, the increased prevalence of dementia, and an increasing need for caregiving, understanding the cognitive health and needs of caregivers to better support them and their care recipients is critical. Unpaid caregivers are an essential facet of a caregiving team; however, these data suggest that caregivers might also need support for their own cognitive and physical health and well-being. Health care professionals can support their patients and their patients’ caregivers by recognizing SCD and its associated challenges in providing care and providing compensatory strategies to promote the health and well-being of both caregivers and their care recipients.^§§^ Public health professionals can continue working to support caregivers and care recipients throughout the caregiving process by strengthening public health infrastructure utilizing the public health strategist approach^¶¶^ and resources such as evidence-based interventions and training materials from CDC’s Building Our Largest Dementia Infrastructure Public Health Center of Excellence on Dementia Caregiving.***

**Summary**

**What is already known about this topic?**

Caregiving can negatively affect caregivers’ physical and mental health. Little is known about caregivers’ cognitive functioning.

**What is added by this report?**

Among unpaid adult caregivers aged ≥45 years, approximately one in eight reported subjective cognitive decline (SCD) (the self-reported experience of worsening confusion or memory loss over the past year). SCD was higher among caregivers (12.6%) than among noncaregivers (10.2%). Caregivers with SCD were more likely than those without SCD to report chronic health conditions, a history of depression, and frequent activity limitations.

**What are the implications for public health practice?**

SCD among caregivers could affect the quality of care provided to care recipients. Health care professionals can support their patients and their patients’ caregivers by recognizing SCD-associated challenges in providing care and providing compensatory strategies to promote the health and well-being of caregivers and their care recipients.

## Footnotes

* https://www.cdc.gov/aging/publications/features/caring-for-yourself.html

† https://www.cdc.gov/brfss/annual_data/2019/pdf/2019-response-rates-table-508.pdf; https://www.cdc.gov/brfss/annual_data/2018/pdf/2018-response-rates-table-508.pdf; https://www.cdc.gov/brfss/annual_data/2017/pdf/2017-response-rates-table-508.pdf; https://www.cdc.gov/brfss/annual_data/2016/pdf/2016_ResponseRates_Table.pdf; https://www.cdc.gov/brfss/annual_data/2015/2015_ResponseRates.html

§ The following 22 U.S. states that included both caregiving and SCD modules in the same survey year during 2015–2019 are included (most recent year used): Alabama (2015), Florida (2015), Hawaii (2017), Illinois (2015), Iowa (2015), Louisiana (2015), Maryland (2019), Mississippi (2015), Missouri (2016), Montana (2016), Nebraska (2015), New Jersey (2018), New York (2019), Oregon (2019), South Carolina (2015), Tennessee (2019), Texas (2019), Utah (2019), Virginia (2019), West Virginia (2015), Wisconsin (2015), and Wyoming (2015).

¶
https://www.cdc.gov/aging/publications/BRFSS-caregiver-brief-508.pdf

** 45 C.F.R. part 46.102(l)(2), 21 C.F.R. part 56; 42 U.S.C. Sect. 241(d); 5 U.S.C. Sect. 552a; 44 U.S.C. Sect. 3501 et seq.

†† Any chronic condition was determined by an affirmative response to the question, “Has a doctor or other health professional ever told you that you had any of the following? For each, tell me Yes, No, or You’re Not Sure: asthma (current); heart attack, angina, or coronary heart disease; a stroke; cancer other than skin cancer; chronic obstructive pulmonary disease, emphysema, or chronic bronchitis; some form of arthritis, rheumatoid arthritis, gout, lupus, or fibromyalgia; kidney disease, not including kidney stones, bladder infections, or incontinence; or diabetes, not including gestational, borderline, or prediabetes?”

§§
https://www.cdc.gov/aging/data/subjective-cognitive-decline-brief.html

¶¶
https://www.cdc.gov/aging/caregiving/caregiver-brief.html
